# Information seeking behavior on hepatitis B virus, and its associated factors among pregnant women at teaching and specialized hospitals, Northwest Ethiopia: A cross-sectional study

**DOI:** 10.1371/journal.pone.0286755

**Published:** 2024-01-22

**Authors:** Adamu Ambachew Shibabaw, Masresha Derese Tegegne, Agmasie Damtew Walle, Sisay Maru Wubante, Nebebe Demis Baykemagn, Melaku Molla Sisay, Adane Nigusie Weldeab

**Affiliations:** 1 Department of Health Informatics, Institute of Public Health, Mettu University, Mettu, Ethiopia; 2 Department of Health Informatics, Institute of Public Health, College of Medicine and Health Sciences, University of Gondar, Gondar, Ethiopia; 3 Department of Health Informatics, School of Public Health, College of Medicine and Health Science, Wollo University, Dessie, Ethiopia; 4 Department of Health Education and Behavioral Science, Institute of Public Health, College of Medicine and Health Sciences, University of Gondar, Gondar, Ethiopia; Debre Berhan University, ETHIOPIA

## Abstract

**Introduction:**

Hepatitis B virus (HBV) infection continues to be a major public health issue worldwide. Health information-seeking behavior is critical to obtain information about health, diseases such as the Hepatitis B virus, health risks, and health promotion and it has become a major concern of health policymakers. However, there is little evidence of information-seeking behavior on the Hepatitis B virus in Ethiopia. So, this study aimed to assess Hepatitis B virus information-seeking behavior and its associated factors among pregnant women at teaching and Specialized Hospitals, in Northwest Ethiopia.

**Methods:**

An institution-based cross-sectional study was conducted among pregnant women at teaching and specialized hospitals, in Northwest Ethiopia from May 01 to June 01, 2022. A total of 423 participants were selected using a systematic random sampling method. The data was collected through an interview-administered questionnaire by kobo-collect software. Then export into SPSS version 20 for analysis. Descriptive statistics, bi-variable, and multivariable logistic regression analyses were done to identify factors associated with Hepatitis B virus information-seeking behavior.

**Results:**

The proportion of information-seeking behavior on the Hepatitis B virus among pregnant women was 40.5% (CI = 35.7, 45.6). Education(diploma and above) [AOR = 3.3, 95% CI (1.31, 8.16)], more than one ANC visit [AOR = 5.99, 95% CI (3.20, 12.31)], smart-phone ownership [AOR = 4.1, 95%CI (1.35, 12.31)], internet access [AOR = 5.1, 95%CI (1.35, 15.60)], perceived susceptibility [AOR = 2.7, 95%CI (1.38, 5.31)], perceived severity [AOR = 3.7, 95%CI (2.06, 6.55)], and self-efficacy [AOR = 1.9, 95%CI (1.03, 3.73)] were factors influencing information seeking on Hepatitis B virus.

**Conclusion:**

The overall proportion of information-seeking behavior on HBV among pregnant women was low. To improve information-seeking behavior on HBV among pregnant women we should connect the women to the internet and technology. Creating women’s awareness about the Hepatitis B virus severity and their venerability and increasing their antenatal care (ANC) visits, self-efficacy, internet access, and women’s education can improve information seeking about the Hepatitis B virus.

## Introduction

Hepatitis B virus (HBV) is a major global public health concern that continues to be a major public health issue globally [2, 3]. One-third of the world’s population (about 2 billion people) has been infected with the Hepatitis B virus at some point in their lives, with 350–400 million chronically infected indeed almost half have acquired their infections either through mother-to-infant transmission or in early childhood, and over 780,000 up to 1.2 million people dying each year [1, 2].

In Sub-Saharan Africa by 2030, with over 20 million people affected and Nigeria has the biggest number of individuals living with Hepatitis B virus (HBV) infection; this infection causes chronic hepatitis, cirrhosis, and hepatocellular cancer [1].

The prevalence of HBV in Ethiopia ranges from 7.4% to 14% (5, 6). It is the second most frequent human carcinogen after cigarettes, and it is one of the primary etiological agents for liver illnesses such as chronic hepatitis, liver cirrhosis, and liver cancer [1]. The virus is highly contagious, with an infectiousness of 100 times that of the human immunodeficiency virus [2]. Hepatitis B is spread by unprotected sexual contact, sharing needles, and syringes, or from mother to baby at birth when blood, sperm, or other body fluids from a person infected with the virus enters the body of someone who is not affected [3]. The information revolution in the health sector is one of the core transformation agendas of the health sector transformation plan of the federal ministry of the Ethiopian government to improve the health status of individuals and the community [3]. Thus, information-seeking behavior is critical to manage HBV infection.

Health information-seeking behavior refers to activities or actions in which people seek information about their health, dangers, illnesses, and health-protective behaviors or health-threatening variables, as well as how to cope with illness, such as searching for, locating, and utilizing disease-related information [1, 2]. Health information seeking and usage are critical for reducing disease morbidity and death and achieving optimal health outcomes [1]. Understanding how pregnant women seek out HBV will help them improve their health and illness prevention [2]. As a result, finding information about HBV is recognized as a life-saving behavior and is used as a preventive strategy, even if the federal ministry of health had implemented the HBV vaccine across risk groups to eradicate HBV and achieve a goal. Hence, information application in the health sector is the government’s concern to improve the health system of the country by managing chronic diseases including HBV. So, Information is the first important element in shifting health-related behaviors and can help patients to cope with health problems [4].

Women of childbearing age can potentially transmit HBV to their babies and they transmit infection to newborns usually during birth or soon after birth following close contact. Newborns who are exposed to HBV will have almost 85–90% risk of developing chronic liver diseases [5, 6]. Pregnant women are a highly venerable group and their babies during birth. As per our literature search, there is little evidence about HBV information seeking and its associated factors among pregnant women in Ethiopia.

This study helped to know the mother’s information-seeking status, behavior, and information source Moreover, the findings of this study will be used as a baseline study by the upcoming researchers. As results from this study assess information seeking about HBV and its associated factors among pregnant women at the teaching and specialized hospitals, in Northwest Ethiopia.

## Methods

### Study design and setting

An institutional-based cross-sectional survey was conducted from May 01 to June 01, 2022, at teaching and specialized hospitals, in North West Ethiopia. The hospital is found in the ancient and historic town of Gondar, northwest Ethiopia, 741 km from Addis Ababa. It is one of the biggest tertiary-level teaching and specialized hospitals in the Amhara Regional State. It provides promotive, preventive, and curative services to over 7 million inhabitants in the catchment area. The hospitals also serves as a research center and provides practical training to medicine and health science students. ANC unit of the specialized referral hospitals is one of the outpatient departments established earlier and currently provides services for about 22,824 pregnant women per year [7].

### Source and study population

All pregnant women attending ANC service at the University of Gondar Specialized and teaching hospital were the Source population. All pregnant women who attended antenatal care services and were available during data collection at the University of Gondar Specialized and teaching hospital were taken as the study population.

### Inclusion and exclusion criteria

In this, all pregnant women attending ANC service at the University of Gondar Specialized and teaching hospital during the data collection were included. All pregnant women attending ANC service at the University of Gondar Specialized and teaching hospital who are unable to hear and speak were excluded from this study.

### Variables of the study

#### Dependent variables

HBV information-seeking behavior (Yes, No)

**Independent variables. Socio-demographic factors:** age, marital status, religion, educational status, occupation, residence, family income, and the number of visits.

**Technological factors:** digital literacy, internet access, Smartphone ownership, and internet satisfaction.

**Behavioral factors:** Alcohol consumption and Smoking.

**Psychological factors:** Perceived susceptibility to HBV, Perceived severity of HBV, and Perceived health self-efficacy of HBV.

**Health-related factors:** health status and health literacy.

### Operational definition

#### Information-seeking behavior about HBV

It is the seeking of information about HBV from any source within the past 9 months before data collection starts. One item question derived from the previous study was used to determine whether respondents sought HBV information in the past 9 months (No = did not seek HBV information in the past 9 months, Yes = seek HBV information within 9 months. Respondents were asked the question have you ever sought information about HBV purposely from different sources in the past 9 months? Subsequently, Respondents were asked about HBV information sources, types of HBV information, and reasons for seeking HBV information [8–10].

### Sample size determination

The sample size was calculated using single population proportion formula with assumptions of 95% confidence level (CI), Z (1-α/2) = 1.96), an expected proportion of HBV information seeking of 50% (p), and a 5% margin of error(d). The total sample size was 423 pregnant women with a 10% non-response rate.

### Sampling procedure

A systematic random sampling technique was used to select study participants from ANC follow-up at Gondar University Specialized and Teaching hospital during the study period. The antenatal care clinic is one of the departments which provides services to 50–70 pregnant women coming from Gondar town and the nearby districts per day, so there are 2, 550 pregnant women served per month. Of the total pregnant women, 423 pregnant women were selected through a systematic random sampling technique. During the data collection period, the interval size of 6^th^ pregnant women was included in the study until the required sample was reached.

### Data collection tools

A structured and Pre-tested interviewer-administered Amharic version questionnaire which was prepared by kobo collect toolbox and adapted from different literature on socio demographic factors [11–13], behavioral factors [14–16], psychological factors [16–18] and health-related factors [9–11, 19] was used to collect the data to make sure that the questionnaires were clear and could be understood by the respondents.

### Data collection procedure

Data were collected by three trained Health informatics professionals. The data collection was supervised by one Health informatics professional, and the selected pregnant women were interviewed through a structured Amharic version questionnaire prepared by the kobo-collect software.

### Data quality control

The questionnaire was translated from English to Amharic (the local language) and then back to English to check its consistency. The data collector and the supervisor were trained for one day on the objective of the study, ethical issues, and study procedure. The tool was pretested in the Felege Hiwot referral hospital which was similar characteristics to the study by taking 5% of the total sample size to check for clarity of language, appropriateness, and internal consistency.

### Data processing and analysis

Data was downloaded from Kobo toolbox and exported into SPSS version 20 for analysis purpose. Each independent variable with dependent (Bivariate) considers the significant variable with the cut point (p-value less than 0.2). Then multivariable logistic regression was used to assess factors associated with HBV information-seeking behavior at a 95% confidence interval P-value; less than 0.05 the variable was significant. Greater than 0.05 for Hosmer and Leme show tests were used to check model fitness which a p-value was 0.975. The results were presented in tables, figures, and text using frequencies and summary statistics such as mean, standard deviation, and percentage to describe the study population about relevant variables. Variables with a P-value less than or equal to 0.05 will be taken as significant.

## Result

### Socio-demographic characteristics of pregnant women

A total of 412 study subjects participated in this study with a 97.4% response rate. The mean age of the study participants was 32.24 ± 7.82 SD years with ranges from 18 to 49 years. The result indicated that around 154(37.4%) study participants were urban residents and 258 (62.6%) were rural residents. Regarding the Educational status of the respondents, 99(24% of the study participants were unable to read and write, 79(19.2%) of the study participants were able to read and write, 77(18.7%) primary education 80(19.4%) secondary education, and 77(18.7%) diploma and above. The result showed that the marital status of 94.4% (389) of study participants was married and 5.6% (21) other (Table 1).

**Table 1:** Socio-demographic characteristics of respondents at teaching and specialized hospitals, Northwest Ethiopia 2022(n = 412).

Variables	Category	Frequency	Percentage (%)
**Residence**	Rural	258	62.6
	Urban	154	37.4
**Age(mean & SD)**		32.24±7.82	
**Number of visits**	One	259	62.9
	More than one	153	37.1
**Religion**	Orthodox	304	73.8
	Muslim	86	20.9
	Other	22	5.3
**Occupation**	Housewife	116	28.2
	Government employee	142	34.5
	Merchant	58	13.1
	Student	65	15.8
	Other	31	7.7
Educational status	Unable to read and write	99	24
	able to read and write	79	19.2
	Primary education(1–8)	77	18.7
	Secondary education(9–12)	80	19.4
	Diploma and above	77	18.7
**Marital status**	Married	389	94.4
	Other	21	5.6
**Monthly income**	<10000	380	92.2
	10000–20000	28	6.8
	> = 25000	4	<5%

**Note:** Others* contain widowed, single, divorced and separated

### Psychological characteristics of pregnant women

Of the total respondents, about 304(73.8%) perceived that they were very concerned about HBV, 208(50.2%) respondents perceived that HBV is a severe disease, and respondents of 296(71.8%) had high confidence about their ability to take good care of their health (Table 2).

**Table 2:** Psychological characteristics to HBV information-seeking behavior at teaching and specialized hospitals, Northwest Ethiopia, 2022(n = 412).

Variable	Category	Frequency	Percentage (%)
Perceived Susceptibility to HBV	Susceptible	304	73.8
	Not Susceptible	108	26.2
Perceived severity of HBV	Sever	208	50.2
	Not sever	204	49.5
**Self-efficacy**	Confident	296	71.8
	Not confident	116	28.2

### Behavioral and health-related characteristics of pregnant women

About 406(98.5%) of the study participants had no history of alcohol drinking and around 403(97.8%) had no history of cigarette smoking. 370(88.6%) of the study participants had no sign and symptoms of HBV. Of the study participants who had signs and symptoms of HBV, and tested for HBV 383(93%) then 45(10.9%) were told by a doctor they had HBV. Regarding current health conditions, about 370(89.8%) of the pregnant women feel healthy only 80(19.4%) of the participants had a family history of HBV (Table 3).

**Table 3:** Behavioral and health-related characteristics to HBV information-seeking behavior at teaching and specialized hospitals, Northwest Ethiopia, 2022(n = 412).

Variables	Category	Frequency	Percentage (%)
Alcohol consumer	No	406	98.5
	Yes	6	4.2
Smoking	No	403	97.8
	Yes	9	3.7
Sign and Symptom	No	365	88.6
	Yes	47	11.4
Perceived Health Status	Feel healthy	370	89.8
	Feel less healthy	42	10.2
Had HBV Test	No	29	7.0
	Yes	383	93.0
HBV history	No	796	95.7
	Yes	36	4.0
**Family HBV history**	No	249	62.4
	Not Sure	77	19.3
	Yes	73	18.5
**Comorbidity**	No	31	91.7
	Yes	380	7.5
**Had HBV**	No	367	89.1
	Yes	45	10.9
Health literacy	Limited	392	95.1
	Adequate	20	5

### Technological characteristics of pregnant women

Of the respondents 148 (35.9%) and 159(38.6%) had smart-phone and internet access respectively. Of the total participants, around 195(49.5%) of respondents had high digital literacy (Table 4).

**Table 4:** Technological characteristics to HBV information-seeking behavior at teaching and specialized hospitals, Northwest Ethiopia, 2022(n = 412).

Variable	Category	Frequency	Percentage (%)
Digital literacy	Low	209	50.7
	High	203	49.5
Smart-phone access	No	258	62.6
	Yes	148	35.9
Internet access	No	253	61.4
	Yes	159	38.6

### Pregnant women information seeking behavior on Hepatitis B virus

**Fig 1:**
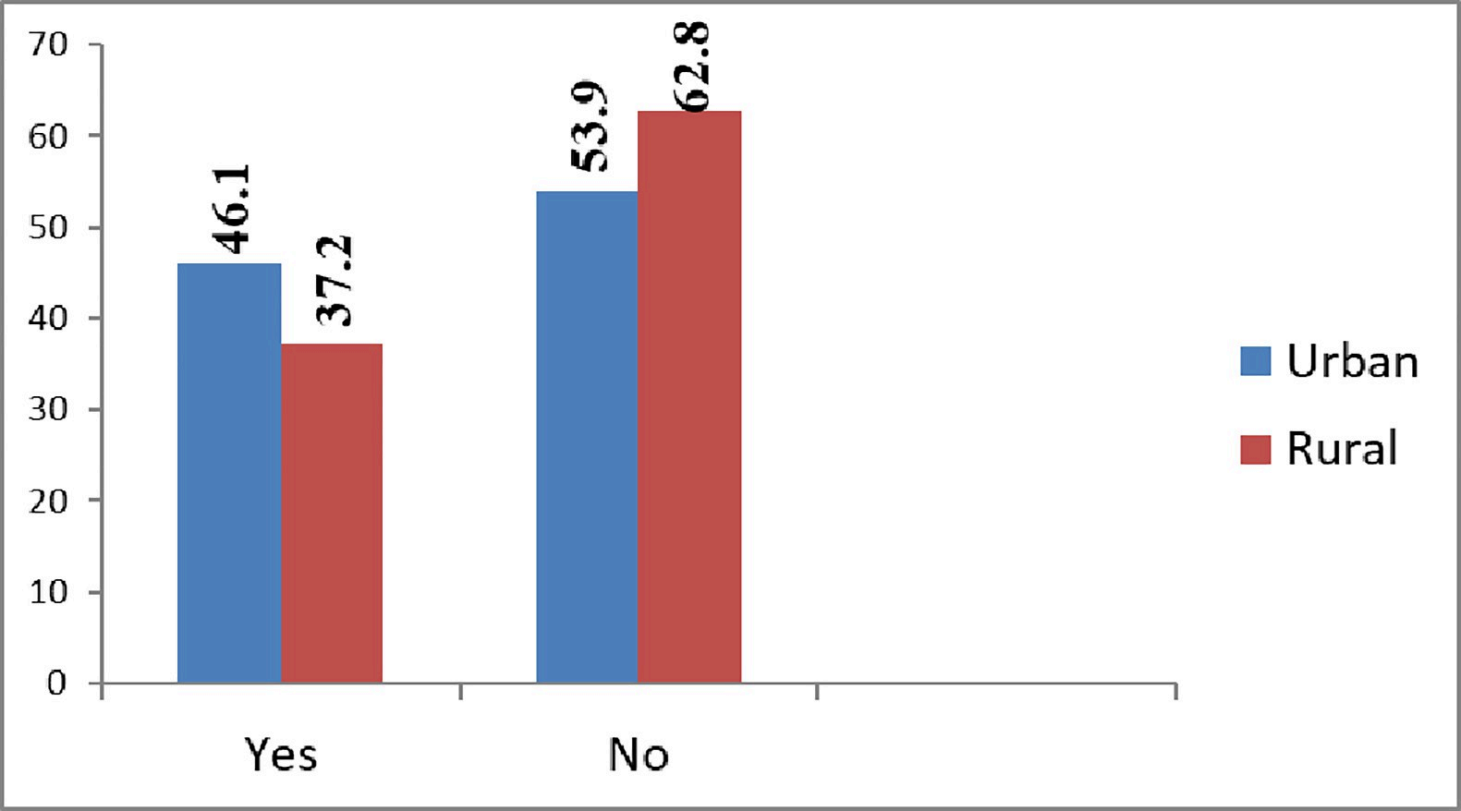
HBV information seeking by pregnant women compared with Urban and rural at teaching and specialized hospitals, 2022.

Of the total respondents, less than half 167(40.5%) of participants had HBV Information-seeking behavior in the past 9 months. Out of the participants, about 153(37.4%) were in the first visit and 259(62.9%) were in the more than visit. 154(32.3%) were rural, and 258(62.6%) were urban residents 66.4% of respondents have used Healthcare providers as primary and the internet 54.3(121) as a secondary source to seek HBV information (Fig 1).

**Fig 2:**
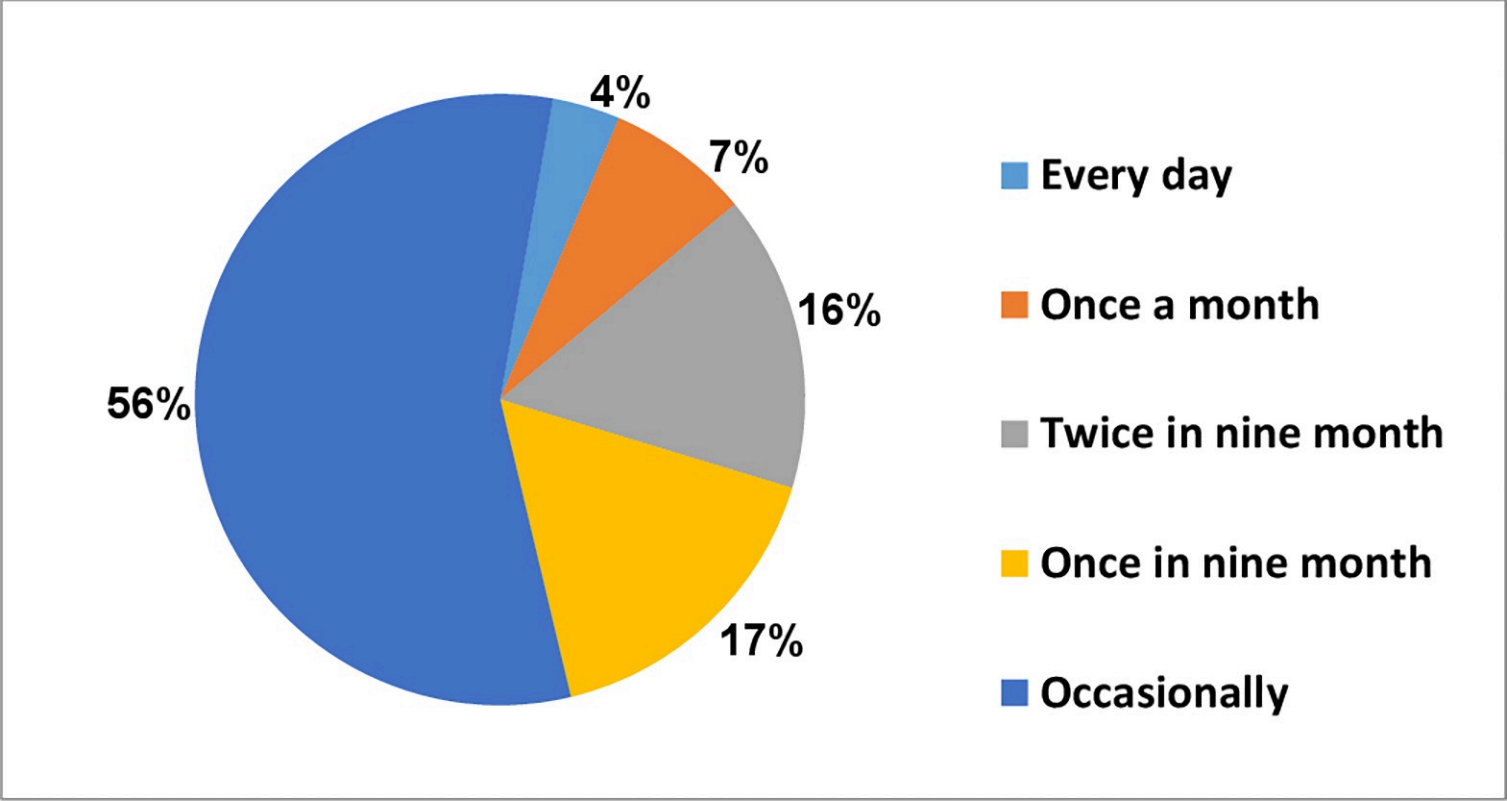
Frequency of HBV information seeking by pregnant women at teaching and specialized hospitals, 2022.

Regarding the frequency, about 56.5% (126) of HBV information seekers sought information occasionally, 16.6% (37) of them sought it once in nine-month, and 15.7% (35) of them sought it twice in nine months. The detailed information about the Frequency of HBV Information seeking is presented in (Fig 2).

**Fig 3:**
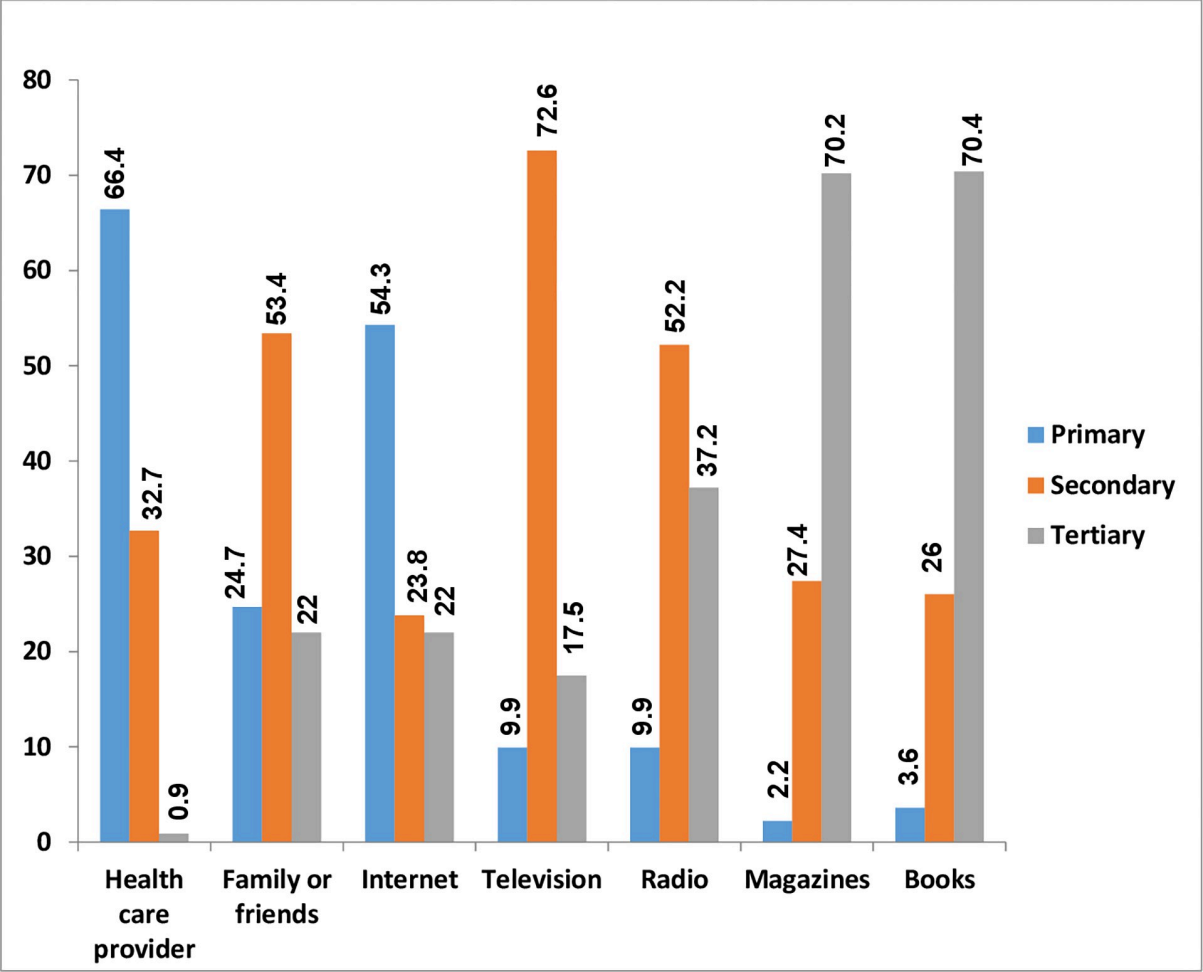
Preferred HBV information source of pregnant women at teaching and specialized hospitals, Northwest Ethiopia 2022.

Out of 167 HBV information seekers, around 88(38.5%) of the respondents sought HBV information for themselves, 30 9(14.0%) of them for their family or friends, and 105(47.5%) of the respondents for both themselves and family or friends (Fig 3).

### Source of information for HBV

Participants were asked about the source of HBV information. The result of this study indicated that 148(66.4%) of HBV information seekers preferred the health care provider as their primary HBV information source followed by the internet 121(54.3%) and next family or friend 55(24.7%) as well as radio/TV 22(9.9%), And Other 13(5.8%) (Table 5).

**Table 5:** Preferred HBV information source of pregnant women at teaching and specialized hospitals, Northwest Ethiopia 2022.

HBV information sources	Primary (%)	Secondary (%)	Tertiary (%)
**Health care provider**	148(66.4)	73(32.7)	4.9
**Family or friends**	55(24.7)	119(53.4)	49(22.0)
**Internet**	121(54.3)	53(23.8)	49(22.0)
**Television**	22(9.9)	162(72.6)	39(17.5)
**Radio**	22(9.9)	118(52.2)	83(37.2)
**Magazines**	4.1	61(27.4)	157(70.2)
**Books**	3.5	58(26.0)	157(70.4)

### Trustable information sources for HBV

The result of this study showed that about 137(63.1%) and 89(39.9%) of the study participants had a lot of trust in HBV information gained from Health care providers and the internet respectively. Others like family or friends 53(23.8%) book 22(9.9%), televisions/radio 18(8.1%), and are also highly trusted HBV information sources by pregnant women. Of the total HBV information seekers, 209(93%) of them had never trusted HBV information from Magazines which has a trust level of 14(6.3%) (Table 6).

**Table 6:** Pregnant women trust different information sources about HBV at teaching and specialized hospitals, Northwest Ethiopia (n = 412).

Trust HBV information sources	A lot (%)	Some (%)	A little (%)	Not At All (%)
**Health care provider**	137(63.1)	70(35.5)	3.7	4.8
**Family or friends**	53(23.8)	124(55.6)	44(19.7)	3.8
**Internet**	89(39.9)	86(38.6)	23(10.3)	25(11.2)
**Television**	18(8.1)	158(70.9)	32(14.3)	15(6.7)
**Radio**	18(8.1)	127(57.0)	69(30.9)	9(4.0)
**Magazines**	14(6.3)	84(37.7)	104(46.6)	21(9.4)
**Books**	22(9.9)	97(43.5)	84(37.7)	20(9.0)

### Reason for seeking information on HBV

The result of this study showed that about 91(40.8%), 71(31.8%), 68(30.5%), 56(25.1%), and 32(14.3%) of the respondent’s primary reason was to sought information about HBV information about HBV treatment, information about HBV prevention, To know HBV sign &symptoms, about HBV diagnosis mechanisms, and global and local burden of HBV respectively (Table 7).

**Table 7:** Reason for seeking information on HBV among pregnant women at teaching and specialized hospitals.

Reasons for Seeking Information on HBV	Primary Reason(%)	Secondary Reason (%)	Tertiary Reason (%)
**For HBV prevention**	71(31.8)	89(39.9)	60(26.9)
**For HBV signs &symptoms**	68(30.5)	121(54.3)	30(13.5)
**For HBV treatment**	91(40.8)	75(33.6)	54(24.2)
**For HBV diagnosis**	56(25.1)	109(48.9)	47(21.1)
**For global and local burden of HBV**	30(13.5)	106(47.5)	55(24.5)

### Reason not seeking information about HBV

Of the total study participants, 245(59.5%) had not sought HBV information from any of the information sources in the last 9 months. The respondents were asked about their reasons for not seeking HBV information. Out of non-HBV information seekers, about 102(53.1%), and 71(37.0%) the respondent’s main reason was Fear of becoming infected with HBV, and HBV information was perceived as irrelevant respectively. Mistrust of online information pregnant women reasons for not seeking HBV information (Table 8).

**Table 8:** Reason not seeking information about HBV among pregnant women at teaching and specialized hospitals (n = 412) 2022.

Reasons for not seeking HBV information	Frequency	Percentage (%)
**Fear about becoming infected with HBV**	102	53.1
**HBV information is perceived as irrelevant**	71	37
**HBV information is not interesting**	17	8.9

### Factors associated with HBV information seeking behavior

To select significant factors associated with HBV information-seeking behavior, bivariate and multivariate logistics regressions were undertaken (Table 9). The result of bivariate logistic regression analysis indicated that age, residence, occupation, educational status, monthly income, number of ANC visit, internet access, smart-phone ownership, Health status, digital literacy, perceived susceptibility, perceived severity, and health self-efficacy were significantly associated with HBV information-seeking behavior. Variables with p-value less than 0.2 were considered for multivariable analysis. In the multivariable analysis educational status (Diploma Higher education), smart-phone ownership, internet access, number of ANC visits, perceived severity, perceived susceptibility, and self-efficacy were significant factors associated with HBV information-seeking behavior. Whereas, age, occupation, family income, residence, occupation, digital literacy, and health status (self-reported health condition) were not significantly associated Model fitness was checked using Hosmer and Lemeshow’s tests and its p-value was 0.975.

**Table 9:** Factors associated with HBV information-seeking behavior among pregnant women at teaching and specialized hospitals (n = 412) 2022.

Variable	Yes (%)	No (%)	COR(95%)		AOR (95%)
Age					
(32.24 ± 7.817 SD)			1.05(1.02,1.08)		1.01(.97,1.05)
Residence					
**Urban**	71(42.5)	83(33.9)	1.44(0.96,2.17)		1.44(0.77,2.70)
**Rural**	96(57.5)	162(66.1)	1		1
Education status					
**Unable to read and write**	49(29.3)	50(20.4)	1		1
**Able to read and write**	40(24.0)	39(15.9)	0.96(0.53,1.73)		0.71(0.31,1.64)
**Primary(1–8)**	39(23.4)	38(15.9)	0.96(0.53,1.73)		0.66(0.27,1.60)
**Secondary(9–12)**	24(14.4)	56(22.9)	2.29(1.23,4.25)		2.25(0.96,5.30)
**Diploma and above**	15(9)	62(25.3)	4.05(2.04,8,06)		**3.27(1.31,8.16)** **
Occupation					
**Housewife**	30(18.0)	97(39.6)	1		1
**GOV’T employed**	85(50.9)	73(29.8)	0.27(0.16,0.45)		0.12(0.10,1.41)
**Student**	22 (13.2)	49(20.0)	0.50(0.17,1,46)		0.52(0.14,1.94)
Follow up					
**One visit**	99(64.7)	54(35.3) 1			1
**More than visit**	68(26.3)	191(73.7)		5.15(3.34,7.93)	**5.99(3.20,12.31)** *
Smartphone					
**Yes**	13(8.1)	148(91.9)		13.97(7.51,25.98)	**4.08(1.35,12.31)** *
**No**	135(55.1)	110(44.9)		1	1
**I**nternet					
**Accessibility**	19(11.4)	140(57.1)		10.39(6.05,17.83)	**5.09(1.76,15.60)** *
**Inaccessibility**	148(88.6)	105(42.9)		1	1
Digital literacy					
**High**	116(57.1)	87(42.9)		4.13(2.71,6.30)	1.44(0.63,3.25)
**Low**	51(24.4)	158(75.6)		1	1
self-efficacy					
**Not confident**	60(51.7)	56(48.3)		1	1
**Confident**	107(36.1)	189(63.9)		1.89(1.23,2.90)	**1.96(1.03,3.73)** **
Perceived-susceptibility					
**Not susceptible**	67(62.0)	41(38.0)		1	1
**Susceptible**	100(32.9)	204(67.1)		3.33(2.11,5.30)	**2.70(1.38,5.31)** **
Perceived severity					
**Not sever**	120(58.8)	84(41.2)		1	1
**Sever**	47(22.6)	161(77.4)		4.89(3.19,7.51)	**3.67(2.06,6.55)** **
Health status					
**Feels healthy**	11(26.2)	31(73.8)		1	1
**Not Feels healthy**	156(42.2)	214(57.8)		2.05(1.00,4.21)	1.10(0.38,3.24)

Note:

*p-value>0.01, p-value<0.01

** and 1 reference category

The odds of HBV information-seeking behavior among pregnant women whose education is a Diploma and above is 3.2 times higher (AOR = 3.27, 95% CI = 1.31, 8.16) than pregnant women who are Unable to read and write. The findings of this study indicated that pregnant women who had ANC visited more than one were 5.9 times more likely to seek HBV information (AOR = 5.99, 95% CI = 3.2, 12.31) when compared to pregnant women who had one ANC visited.

In addition to this, the participant who had high perceived severity of HBV were 3.6 times more likely to seek HBV information (AOR = 3.67, 95% CI = 2.06.,6.54) compared to participants who had low perceived severity of HBV. The participant who had high perceived susceptibility to HBV were 2.7 times more likely to seek information (AOR = 2.70, 95% CI = 1.38, 5.31) compared to participants who had low perceived susceptibility to HBV. The participant who had high health self-efficacy to HBV were 1.9 times more likely to seek information (AOR = 1.96, 95% CI = 1.03, 3.73) compared to participants who had low health self-efficacy to HBV.

## Discussion

This study tried to find out pregnant women’s information-seeking behaviors about HBV. The result from this study is used to identify the possible HBV information sources preferred by pregnant women and factors associated with HBV information-seeking behavior. In addition to this, the findings will give directions to the pregnant women to use different HBV information sources to prevent themselves from HBV. This revealed that the majority of pregnant have not sought information about HBV to prevent themselves.

Findings of the current study indicated that 40.5% (CI = 35.7, 45.6) of the pregnant women sought HBV information from different sources in the past 9 months, which is consistent with a study conducted in Ethiopia Bahirdar city public hospitals (44.0%) [20]. And Debre Markos referral hospital (41.6). However, this study is lower than another study done at Ethiopia University of Gondar (55.5) [21]. This variation might be due to the differences in educational status because the previous study was on the University student. Similarly, lower than another studies done in Malaysia (62.4%) [22], Indonesia 57.9% [17] and Iran 75.2% [23]. This variation might be due to the differences in technological advancement and IT infrastructure. The possible explanation for this discrepancy in the result might be due to the low information communication development index and internet penetration in Ethiopia, according to the 2021 World Internet Statistics report, the internet penetration rate in Ethiopia is 17.9% [20].

Regarding HBV information sources, around 66.4% of HBV information seekers preferred the health care provider as their primary HBV information source followed by 54.3% from the internet 54.3%, which is consistent with the previous study conducted In Ethiopia, in which 48% of respondents preferred health care provider as the main health information source and 28% from the internet as the second source of health information [24]. The study also varies with the previous study which stated that the internet was the main source of health information with 56.9% of information seekers preferring the internet as their primary information source the respondents preferred the Internet as their primary health information source [21]. This variation could be due to the previous study done on the students. Another possible reason for the differences might be the lack of pregnant women’s internet skill for the importance of HBV information sources for their health [25].

The study indicated that pregnant women who have ANC visited more than one was more likely to seek information, as compared to pregnant women, were have one ANC visit. The possible reason for this is that pregnant women who have more than one visit had high awareness about the transmission and prevention mechanisms of HBV due to a every visit counseling [10]. Pregnant women whose level of education was a diploma and above were more likely to seek information as compared to unable to read and write. This result is in line with the previous study conducted in Ethiopia [26] which respondents with lower educational attainment were less likely to seek health information but higher education attainment more likely [25]. This might be due to that the more educated pregnant women, the more knowledge they acquire about the impact of HBV on their health and this enables the pregnant women to seek HBV information.

In this study respondents who had internet access were more likely to seek HBV information when compared to those who had limited internet access. This result is consistent with a study conducted in Ghana [27]. And a study conducted in Ethiopia [21]. The possible explanation for this could be internet is the main preferred for health information seeking and social media use and due internet technology advancement [19]. This study has also found that ownership of a smart-phone was 4.1 times more likely to seek HBV information compared to those who hadn’t a smart-phone. This finding is supported by a study conducted in Ethiopia smart-phone has significance for the utilization of health information and smart-phone is the current enabler for searching electronic information [28].

This study showed that participants who had high perceived severity of HBV were more likely to seek HBV information than respondents who had low perceived severity of HBV. This finding is consistent with another study conducted in Malaysia [29]. The belief that HBV causes a serious impact on their health may encourage the action of HBV preventive behavior if pregnant women feel threatened by the infection [24]. Individuals who perceive HBV as severe want to know the risk factors, prevention mechanisms, diagnosis, and treatment of HBV [30].

In the current study, perceived susceptibility to HBV was significantly associated with HBV information-seeking behavior. Respondents who were concerned about getting HBV were more likely to seek HBV information than those who were not concerned at all about getting HBV. This result is consistent with a previous study conducted in Ethiopia [21] and a study conducted in Malaysia [20]. The possible explanation might be the perceived susceptibility to health problems had a positive impact on Internet use for seeking health information and this led to a decrease in the burden of HBV pregnant women thinks they were more venerable to HBV they seek about HBV [29].

Furthermore, Self-efficacy was also found to be a factor that was significantly associated with information-seeking about HBV. Thus respondents who were confident to execute or undertake the behavior to reduce the risk of developing HBV were 1.9 times more likely to seek information about HBV when compared with respondents who were not confident to take behavior or actions to reduce the risk of developing HBV. This result is consistent with a previous study conducted in Ethiopia [21]. The possible explanation might be self-efficacy to health problems had a positive impact on Internet use for seeking health information and this led to reducing the burden of HBV If pregnant women were more confident about their health so they motivated to seek about HBV [30].

### Strengths and limitations of the study

As to my literature search, the study is the first attempt in the area so future researchers should use the results as baseline data. The study was a facility-based cross-sectional study which may not articulate to us the causal inference between variables. The study assessed HBV information-seeking behavior in the past 9 months, there may have been a chance of recall bias by the participants. Interviewer-administered data collection methods may be affected by Interviewer bias.

## Conclusion

In general, HBV information-seeking behavior among pregnant women was low. The findings indicated that the most trusted HBV information by pregnant women was information gained from Health care providers and the Internet respectively. Educational status (diploma and above), pregnant women reached in more than one visit, Internet access, smart-phone ownership, health self-efficacy, Perceived susceptibility, and perceived severity of HBV were significant factors associated with HBV information-seeking behavior. Focusing on establishing an internet connection, helps them to improve self-efficacy pregnant women’s education, awareness about ANC importance, venerability and the danger of HBV could improve the HBV information-seeking behavior of pregnant women.

## Supporting information

S1 Data (ZIP)

## Abbreviations and acronyms

ANC: Anti-natal care
AOR: Adjusted odd ratio
CI: confidence interval
FMOH: Federal Minister of Health
HBV: Hepatitis B virus
HSTP: Health Sector Transformation Plan
IT: Information technology
SPSS: Statistical Package for the Social Sciences
WHO: World Health Organization
